# Supplementary material for: Effects of short-term pre-competition weight loss on certain physiological parameters and strength change in elite boxers
Source: PLoS One. 2024 May 23;19(5):e0304267. doi: 10.1371/journal.pone.0304267 (PMC11115213; doi:10.1371/journal.pone.0304267)
Supplement: Attachment — Submitted filename: Response to Reviewers (1).docx [file pone.0304267.s002.docx]

Academic Editor

About Journal Requirements:

1.	Please ensure that your manuscript meets PLOS ONE's style requirements, including those for file naming. The PLOS ONE style templates can be found at 
<https://journals.plos.org/plosone/s/file?id=wjVg/PLOSOne_formatting_sample_main_body.pdf> and 
<https://journals.plos.org/plosone/s/file?id=ba62/PLOSOne_formatting_sample_title_authors_affiliations.pdf>

Answer: updated, thank you!

Please provide additional details regarding participant consent. In the ethics statement in the Methods and online submission information, please ensure that you have specified (1) whether consent was informed and (2) what type you obtained (for instance, written or verbal, and if verbal, how it was documented and witnessed). If your study included minors, state whether you obtained consent from parents or guardians. If the need for consent was waived by the ethics committee, please include this information. If you are reporting a retrospective study of medical records or archived samples, please ensure that you have discussed whether all data were fully anonymized before you accessed them and/or whether the IRB or ethics committee waived the requirement for informed consent. If patients provided informed written consent to have data from their medical records used in research, please include this information.

Answer: Dear Editor, Our research did not involve under-aged children, so we did not need to obtain parental consent. The process was organised with a consent form in which all data will be completely anonymised and the ethics committee was informed before accessing the data. Informed written consent was obtained from the participants for the use of the data in their records in the research. Also separate written consent form was obtained for each group and the method section (study design) was presented.

3. Your ethics statement should only appear in the Methods section of your manuscript. If your ethics statement is written in any section besides the Methods, please move it to the Methods section and delete it from any other section. Please ensure that your ethics statement is included in your manuscript, as the ethics statement entered into the online submission form will not be published alongside your manuscript.

Answer: Dear Editor, we have presented the ethical statement of the research in the method section. As you have stated, we have not re-presented the information about our ethical statement in any other section.

4. Please include captions for your Supporting Information files at the end of your manuscript, and update any in-text citations to match accordingly. Please see our Supporting Information guidelines for more information: <http://journals.plos.org/plosone/s/supporting-information.>

Answer: updated, thank you!

5. We notice that your supplementary figures are uploaded with the file type 'Other'. Please amend the file type to 'Supporting Information'. Please ensure that each Supporting Information file has a legend listed in the manuscript after the references list.

Answer: updated, thank you!

6. Please review your reference list to ensure that it is complete and correct. If you have cited papers that have been retracted, please include the rationale for doing so in the manuscript text, or remove these references and replace them with relevant current references. Any changes to the reference list should be mentioned in the rebuttal letter that accompanies your revised manuscript. If you need to cite a retracted article, indicate the article’s retracted status in the References list and also include a citation and full reference for the retraction notice.

Answer: Dear Editor, we have reviewed our reference list according to your journal rules and tried to update the references that were found to be inappropriate and incorrect, and tried to show the changes made with the red font "Revised Manuscript with Track Changes’’. In addition, the references have not been replaced and no references have been removed from the main text.

Reviewer #1:

1. It would be useful for practice if the authors gave us an answer to the following: Is the strength they possess after the experimental program still higher than those who do not remove weight?

Answer: Thanks for your professional review work on our article. We agree with your point of view but there were no possibilities to follow the boxers' achievements in the championships. We did not have authorisation for this. Therefore we avoided the statement that it definitely increased the athletes' competition performance. In addition, one of the important limitations of the research included a follow-up and evaluation before the championships. However, we were able to report some of our observations about the change in performance. Although there was no statistically significant difference between the C group and the E+D1 group, quadriceps submaximal and maximal strength development and hamstring submaximal and maximal strength development of the E+D1 group were higher than the C group. Similar situation was observed in shoulder internal and external rotators as well as arms and legs measurements. This situation suggests that especially for boxers, rapid weight loss may be a disadvantage for them, on the contrary, they may prevent loss of strength in a planned program with adequate caloric intake and protein support instead of short-term dietary restrictions while dropping weight. Therefore, we consider this research to be important in terms of demonstrating the value of controlling the training and nutrition periods of boxers with adequate calorie consumption and protein intake.

Reviewer #2:

1. Exclude abbreviation in abstract that are not well-known e.g. describe the groups of boxers clearly etc;

Answer: Thanks for your professional review work on our article. We agree with your point of view, we've describe the groups of boxers and abstract section accordingly, corresponding changes have been highlighted in red in the revised version.

2. Do not put unnecessary information in abstract as the abstract has to contain just the most important information that will persuade potential readers to read the whole manuscript e.g. “The data obtained were analyzed in SPSS 22.0 package software” – this sentence is absolutely unnecessary etc.;

Answer: Thank you for your generous comments. We very much valuable your view that "The summary should contain only the most important information, do not put unnecessary information in the summary". We have made the appropriate changes and they are highlighted in red in the revised text.

3.	The method section is too long and it is hard to read the manuscript, so I recommend to use some protocol that is already published and just cite it with some highlighted modifications

Answer: Thank you very much for your professional comments about the method. You are correct that it is long and difficult to read. Considering your valuable guidance, we have tried to present the diet and exercise part with a summarising figure and presented the protocols that we have taken as reference. We have tried to present the diet content and exercise method as in the research, since we have not come across such an approach and research on boxers before, and also in order to follow the effects of the boxers' pre-competition nutrition and exercise intensity.

4.  	From the reason the limitations are mentioned but did not explained briefly, I do believe it is necessary to add recommendations for the further studies and share some experiences from this study to help researchers who will replicate this study in setting up the study designed and avoid some eventual methodological issues;

Answer: Thanks so much for your useful comments. We apologize for the confusion caused by our unclear expression. Thanks to you, we have recognised our shortcoming regarding limitations and have done our best to overcome it and make suggestions for future research.

5.	The reference list should be revised and updated by full meta data.

Answer: We are thankful that we have updated the deficiencies in the reference list, which we have overlooked and which you are absolutely right, and presented them again for your evaluation.

Thank you!
