# Supplementary material for: Effects of short-term pre-competition weight loss on certain physiological parameters and strength change in elite boxers
Source: PLoS One. 2024 May 23;19(5):e0304267. doi: 10.1371/journal.pone.0304267 (PMC11115213; doi:10.1371/journal.pone.0304267)
Supplement: Attachment — Submitted filename: rebuttal letter.docx [file pone.0304267.s003.docx]

**Reviewer #3:** very good study. the authors have decided on a topic that is very interesting and current. I have to praise the methodological setting of the work, because the method of research is presented very clearly and in detail.
my only objection is of a technical nature:
- when you show figure 1, you put its title above the graph, and for the others (figure 2, figure 3...) you put the title below the graph. It must be identical, as prescribed by the standard.

Response: Adjusted, thank you very much.

Best regards!
